# Na^+^, K^+^-ATPase: Ubiquitous Multifunctional Transmembrane Protein and its Relevance to Various Pathophysiological Conditions

**DOI:** 10.4021/jocmr2010.02.263w

**Published:** 2010-02-26

**Authors:** Mohd Suhail

**Affiliations:** a Department of Biochemistry, University of Allahabad, Allahabad-211002, India

## Abstract

The Na^+^, K^+^-ATPase (NKA) is an ubiquitous enzyme consisting of α, β and γ subunits, and is responsible for the creation and maintenance of the Na^+^ and K^+^ gradients across the cell membrane by transporting 3 Na^+^ out and 2 K^+^ into the cell. Sodium pump regulation is tissue as well as isoform specific. Intracellular messengers differentially regulate the activity of the individual NKA isozymes. Regulation of specific NKA isozymes gives cells the ability to precisely coordinate NKA activity to their physiological requirements. It is the only known receptor for the cardiac glycosides used to treat congestive heart failure and cardiac arrhythmias. Endogenous ligands structurally similar to cardiac glycosides may act as natural regulators of the sodium pump in heart and other tissues. Identification of naturally occurring regulators of  NKA could initiate the discovery of new hormone-like control systems involved in the etiology of selected disease processes, hence the importance of understanding the relation of the sodium pump and its ligands to disease. Diabetes has a marked effect on the metabolism of a variety of tissues and because the NKA is critical for the membrane potential and many transports, a change in its activity in diabetes would have profound consequence in these tissues. NKA is also involved in hypertension, salt balance, cardiovascular and renal disorders, sperm capacitation, cell volume regulation, apoptosis, rheumatoid arthritis, sepsis, neurological disorders, lung edema clearance and preeclampsia. NKA activity and expression in the collecting duct of kidney are modulated physiologically by hormones like aldosterone, vasopressin, and insulin. NKA enzyme activity and subunit levels are reduced in carcinoma, NKA-β levels were highly reduced in an invasive form of human renal clear cell carcinoma, androgen-dependent prostate cancer, in early stages of urothelial cancer, as well as in poorly differentiated, highly motile carcinoma cell lines obtained from various tissues suggesting a functional link between reduced NKA-β expression and cancer progression. It could be a target for the development of anticancer drugs as it serves as a signal transducer, it is a player in cell adhesion and its aberrant expression and activity are implicated in the development and progression of different cancers.

**Keywords:**

Na^+^, K^+^-ATPase (NKA); Cardiotonic steroids (CTS); Diabetes; Hypertension; Cardiovascular and renal disorders; Signal transducer; Anticancer drugs

## Introduction

The Na^+^, K^+^-ATPase (NKA) is an ubiquitous enzyme consisting of α, β and γ subunits, and is responsible for the creation and maintenance of the Na^+^ and K^+^ gradients across the cell membrane by transporting 3 Na^+^ out and 2 K^+^ into the cell. Most living cells have a high concentration of intracellular K^+^ and a low concentration of intracellular Na^+^. This is in contrast to the outside of the cell, where there is a high concentration of extracellular Na^+^ and a low concentration of extracellular K^+^. Thus, a concentration gradient exists for the loss and gain of intracellular K^+^ and Na^+^, respectively. This gradient is maintained through the activity of various ionic channels and transporters, but predominantly the activity of the NKA. A typical cell keeps a resting membrane potential of -70 mV. Potassium ions will tend to flow out of the cell, since their equilibrium potential (-91 mV) is more negative than the transmembrane potential. Sodium ions have a very strong force driving them into the cell, since both the chemical and electrical gradients (equilibrium potential of +64 mV) favor Na^+^ uptake. The enzymatic manifestation of the sodium pump is the NKA. This enzyme, found in all mammalian cell membranes, is necessary for proper cellular function since it helps to preserve the ionic gradients across the cell membrane and thus the membrane potential and osmotic equilibrium of the cell [1]. This function is crucial for cell survival and body homeostasis since the Na^+^ gradient is used as an energy source to transport ions or solutes, and is at the origin of the vectorial Na^+^ reabsorption in the kidney and of action potentials in excitable tissues. It is putative component of a biological membrane that affects the transfer of these ions from one side of the membrane to the other. It is active transport which is responsible for the well-established observation that cells contain relatively high concentrations of potassium ions but low concentrations of sodium ions. The mechanism responsible for this is the sodium-potassium pump which moves these two ions in opposite directions across the plasma membrane. This was investigated by following the passage of radioactively labeled ions across the plasma membrane of certain cells. It was found that the concentrations of sodium and potassium ions on the two other sides of the membrane are interdependent, suggesting that the same carrier transports both ions. It is now known that the carrier is an ATPase and that it pumps three sodium ions out of the cell for every two potassium ions pumped in. It catalyzes the transfer of 2 K^+^ from the extracellular space into the cell and the extrusion of 3 Na^+^, while hydrolyzing adenosine triphosphate (ATP) to adenosine diphosphate (ADP) and inorganic phosphate (Pi). The transport of 3 Na^+^ for 2 K^+^ across the membrane, through the means of the sodium pump, maintains transmembrane gradients for the ions and produces a convenient driving force for the secondary transport of metabolic substrates such as amino acids and glucose. In addition, the nonequivalent transport is electrogenic and leads to the generation of a transmembrane electrical potential allowing cells to become excitable. The “pump” couples the energy released in the intracellular hydrolysis of adenosine triphosphate (ATP) to the transport of cellular ions, a major pathway for the controlled translocation of sodium and potassium ions across the cell membrane. NKA therefore controls directly or indirectly many essential cellular functions, e.g., cell volume, free calcium concentration, and membrane potential. Regulation of this enzyme (transporter) and its individual isoforms is thought to play a key role in the etiology of some pathological processes.

The transport ATPases were reported in 1957 by a Danish scientist named J.C. Skou [2]. This was the first report suggesting that Na^+^ and K^+^ transport across the plasma membrane is linked to activation of NKA (otherwise known as sodium pump). Forty years later, in 1997, J.C. Skou shared the Nobel Prize in Chemistry for his discovery of NKA. Transport ATPases have been classified into three categories: (1) P-type ATPases that catalyze reactions using a phosphorylated intermediate; (2) V-type ATPases that are found to be associated with vacuoles; and (3) F-type ATPases that are also known as ATP synthases. The primary role of the P-type NKA is to maintain the homeostasis of Na^+^ and K^+^ ions in eukaryotic cells.

The purpose of this review is to highlight the structure of NKA and the relevant literature information showing its involvement in various patho-physiological processes, which may help others for further exploration to control diseases where NKA is involved.

**Figure 1:**
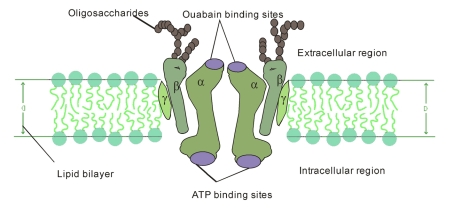
Diagrammatic representation of NKA consisting of 2α, 2β, 2γ-subunits within the biomembrane.

The NKA contains 1 principal catalytic subunit, designated α and 1 sugar-rich auxiliary subunit, designated β. There is an associated subunit γ present only in some tissues. The α-subunit has a molecular mass of about 110 kDa with 10 transmembrane segments. Its four distinct isoforms have been identified. The differences of amino acid sequences among the isoforms are minor. They are each coded by a different gene, some of them located on different chromosomes [3,4]. The various isoforms differ primarily in their tissue distribution, α1 predominating in several tissues, including kidney, nerves, and lung; α2 in skeletal muscle and heart; α3 in the brain; and α4, which is apparently localized to testis and specifically to spermatozoa [5]. The α-subunit carries the catalytic function of the enzyme, and this is reflected in its possession of several binding and functional domains. The β-subunit has a molecular weight of about 55 kDa, with a single membrane crossing. Its three isoforms have been identified. As α isoforms, β isoforms have a tissue-specific distribution, β1 is ubiquitous, β2 is expressed in skeletal muscle and heart, and β3 in testis and central nervous system [6]. It is clear that an essential role for β subunit is in the delivery and the appropriate insertion of α subunit in the membrane [7]. In recent years, a variety of studies suggest that the β subunit may be more intimately involved in the mechanism of active transport and may be a regulatory subunit [5,8]. The γ subunit is a hydrophobic and a single-membrane crossing protein of molecular weight about 12 kDa (Fig. 1).

Although much is not known about its function, it does appear to be obligatorily associated with the αβ complex [9]. Further, the other family of small membrane proteins i.e. FXYD proteins exist widely distributed in mammalian tissues with prominent expression in tissues that perform fluid and solute transport or that are electrically excitable. The FXYD protein family is a family of small membrane proteins that share a 35-amino acid signature sequence domain, beginning with the sequence PFXYD and containing 7 invariant and 6 highly conserved amino acids. The approved human gene nomenclature for the family is FXYD-domain containing ion transport regulator. Recent experimental evidence suggests that at least five of the seven members of this family, FXYD1 (phospholemman), FXYD2 (γ-subunit of NKA), FXYD3 (Mat-8), FXYD4 (CHIF), and FXYD7, are auxiliary subunits of NKA and regulate NKA activity in a tissue. These results highlight the complexity of the regulation of Na^+^ and K^+^ handling by NKA, which is necessary to ensure appropriate tissue functions such as renal Na^+^ reabsorption, muscle contractility, and neuronal excitability. Moreover, a mutation in FXYD2 has been linked to cases of human hypomagnesemia, indicating that perturbations in the regulation of NKA by FXYD proteins may be critically involved in pathophysiological states [10].

The γ-subunit is a member of the membrane protein family that consists of proteins having only one transmembrane domain. Expressed in oocytes, such proteins serve as ion channels [11]. Further, that the addition of γ-subunit to NKA changes its affinity to monovalent cations: the α1β1γ complex has lower affinity to Na^+^ and K^+^ than the α1β1 complex [12]. In addition, there is apparently a family of proteins that are relatives (isoforms) of this subunit: two of them found in rat kidney have seven different amino acid residues in the N terminal domain, and one of these two forms can be acylated [13]. Isoforms of the γ-subunit have different electrophoretic mobility and differently affect the affinity of the α1β1γ complex for Na^+^ [12,13]. These data suggest that the γ-subunit in kidney is a factor regulating the sensitivity of NKA to transported cations.

In 2005, Capasso et al [14] have reported that hypertonicity-mediated upregulation of the γ-subunit of NKA is dependent on both the c-Jun NH2-terminal kinase (JNK) and the phosphoinositide 3-kinase (PI3 kinase) pathways. They explored the mechanisms whereby these pathways regulate the expression of the γ-subunit in inner medullary collecting duct cells (IMCD3). Inhibition of JNK with SP-600125 (20 mM, an anthrapyrazolone inhibitor of JNK), a concentration that causes an approximately 95% inhibition of hypertonicity-stimulated JNK activation, markedly decreased the amount of the γ-subunit in response to 550 mosmol/kg H2O for 48 h. This was accompanied by a parallel decrease in the γ-subunit mRNA. The rate at which the γ-subunit mRNA decreased was unaffected by actinomycin D. In contrast, inhibition of PI3 kinase with LY-294002 (a selective PI3K inhibitor) results in a marked decrease in the amount of γ-subunit protein but without alteration in γ-subunit message. The rate at which the γ-subunit protein decreased was unaffected by cyclohexamide. Transfection of IMCD3 cells with a γ-subunit construct results in the expression of both γ-subunit message and protein. However, in cortical collecting duct cells (M1 cells) such transfection resulted in expression of only the message and not the protein. They concluded that JNK regulates the γ-subunit at the transcriptional level while PI3 kinase regulates γ-subunit expression at the translational level. There is also post-transcriptional cell specificity in the expression of the γ-subunit of NKA.

The γ-subunit has been characterized as a fine-tuning modulator of the NKA expressed in kidney tissues. This small single transmembrane domain protein interacts with the γ-subunit of NKA to increase affinity for ATP and decrease affinity for Na^+^ allowing medullary cells to continue pump activity under reduced cellular ATP levels. The γ-subunit is undetectable in kidney cell cultures grown under isotonic conditions and expression is induced with acute or chronic exposure to hypertonicity. The gamma subunit demonstrates remarkable regulatory complexity including induction by chloride ions rather than sodium, the differential expression of at least 2 isoforms, involvement of separate MAP (mitogen-activated protein) kinase signaling pathways for transcription (JNK) and translation (PI3K) as well as cell type regulation of expression. Mutation in the transmembrane domain of the γ-subunit has been implicated in cases of primary hypomagnesemia. Alternative roles have been established for the γ-subunit in embryonic development and quite possibly additional functions in cell signaling as yet unrecognized may be possible [15].

**Figure 2:**
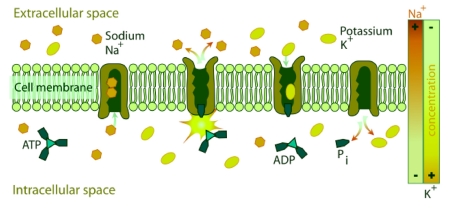
Diagrammatic representation of the transport of Na^+^[3] and K^+^[2] by NKA across the biomembrane consuming one molecule of ATP.

NKA couples the energy released in the intracellular hydrolysis of ATP to the export of three intracellular sodium ions and the import of two extracellular potassium ions. The continuous operation of this macromolecular machine ensures the generation and maintenance of concentration gradients of sodium and potassium across the cell membrane (Fig. 2). This electrochemical gradient provides energy for the membrane transport of metabolites, nutrients, and ions. This electrochemical gradient is essential also for regulation of cell volume and intracellular pH and for the action potential of muscle and nerve [9]. This enzyme is responsible of about 15% to 20% of resting energetic expense in whole organism [16].

Because several cellular transport systems are coupled to the movement of sodium and, therefore, to the function of NKA, this enzyme is the target of multiple regulatory mechanisms activated in response to changing cellular requirements. The demand for modulators of the NKA is likely to be greatest in tissues in which disturbances of the intracellular alkali cation concentration underlie their specialized functions. Prime examples are the changes in enzyme activity that occur in response to physiological stimuli such as nerve impulse propagation and exercise [9]. Generally, regulation of NKA is brought about by increased message transcription, increased recruitment of heterodimers to the cell membrane, modifications of heterodimers trafficking, and half-life in the cell membrane, and by direct regulation of enzymes in the cell membrane. Direct regulation of the cell membrane enzymes results from phosphorylation and dephosphorylation by protein kinases and protein phosphatases. Thus, depending on the tissue, activation of protein kinases can induce an increase or decrease in sodium pump activity [17]. Moreover, sodium pump regulation seems to be tissue but also isoform specific [18]. Firstly, the simplest and most straightforward determinants of pump activity are the concentrations of substrates- Na^+^, K^+^, and ATP. Some hormones appear to alter NKA activity by modifying its apparent affinity for sodium or by enhancing the sodium influx. The ATP concentration is generally saturating for the enzyme in most cells. However, in some tissues and under certain conditions, ATP levels may go down to sub-saturating levels, such as in kidney medulla, which functions under near anoxic conditions [19]. Secondly, endogenous cardiotonic steroids such as ouabain inhibits specifically the sodium pump, whereas interactions of the pump with components of the cytoskeleton permits the correct processing and targeting of sodium pumps to the appropriate membrane compartment [9]. Thirdly, NKA is a transmembranous enzyme and many reports have focused on the role of membrane lipids. In general, lipids that promote bilayer formation of physiological thickness and increased fluidity tend to support optimal NKA activity, as do negatively charged lipids such as phosphatidylserine and phosphatidylglycerol [20-22]. The effects of cholesterol on enzyme activity are often also related to membrane fluidity [23]. Free fatty acids present in the membranes or as the products of phospholipases and eicosanoids tend to have various regulatory effects on NKA activity. Lastly, the enzyme activity is subjected to both short- and long-term regulation by several hormones. Short-run regulation involves generally direct effects on the kinetic behavior of the enzyme or translocation of sodium pumps between intracellular stores and the plasma membrane. Long-run regulation induces de novo NKA synthesis or degradation. Corticosteroids and particularly aldosterone sustains a long-run increase in expression of sodium pumps, whereas catecholamines have various affects on pump activity, with an inhibitory effect of dopamine and a stimulating effect of epinephrine and norepinephrine [9]. Insulin mainly stimulates the NKA activity by increasing the translocation of sodium pumps from intracellular stores to the cell surface, the cytoplasmic sodium content, and also the apparent affinity of the enzyme for sodium [24]. Recently, C-peptide (a peptide that is made when proinsulin is split into insulin and C-peptide; –one C-peptide for each insulin molecule) has been found to stimulate the NKA activity in renal tubular from control rat [25] and in sciatic nerve from diabetic rat [26]. In human diabetes, the decrease of availability of both insulin and C-peptide could change the regulatory equilibrium of NKA activity in favor of a decrease.

## Isoenzymic forms of NKA

As mentioned above the NKA is characterized by a complex molecular heterogeneity that results from the expression and differential association of multiple isoforms of both its α- and α-subunits. At present, as many as four different α-polypeptides (α1, α2, α3, and α4) and three distinct β-isoforms (β1, β2, and β3) have been identified in mammalian cells. The stringent constraints on the structure of the sodium pump isozymes during evolution and their tissues specific and developmental pattern of expression suggests that the different NKA have evolved distinct properties to respond to cellular requirements. The kinetic characteristics of different NKA isozymes to the activating cations (Na^+^ and K^+^), the substrate ATP, and the inhibitors Ca^2+^ and ouabain demonstrate that each isoform has distinct properties. In addition, intracellular messengers differentially regulate the activity of the individual NKA isozymes. Thus, the regulation of specific sodium pump isozymes gives cells the ability to precisely coordinate NKA activity to their physiological requirements [27].

## Pathophysiological relevance of NKA

### Alterations in NKA activity in diabetes

Diabetes has a marked effect on the metabolism of a variety of tissues and because the NKA is critical for the membrane potential and many transports, a change in its activity in diabetes would have profound consequence in these tissues. We have observed significant effect of diabetes in the metabolism of diabetic erythrocytes [28-33] and significant decrease in the activity of NKA in alloxan induced diabetic rats [30]. Erythrocytes of diabetic patients have reduced life span [28], altered membrane dynamic properties and increased membrane thermostability. It has also been reported that diabetic patients with poor metabolic control have lower erythrocyte membrane enzymes activity as compared to healthy control subjects [30-32]. The modified proteins have altered functions such as modified enzymatic activities [29-33], lower affinities for their receptors [34], etc. In 1976, Das et al [35] described a decrease of NKA enzyme activity in sciatic nerve of diabetic rat, whereas an increase in enzyme activity was found in mucosa of the small intestine of diabetic rat [36]. These two examples illustrate the different effect of diabetes on NKA depending on the tissues. Tissues can be classified in three groups, one principal group in which diabetes induces a decrease in NKA activity, including sciatic nerve, lens, heart, and erythrocyte; another group in which diabetes causes an increase in enzyme activity, including mucosa of the small intestine; and one group with a NKA activity unchanged or where it exists differences between studies.

Several mechanisms have been suggested to explain the decrease in NKA activity: a depletion of the intracellular pool of myo-inositol, an increased flux through the aldose reductase pathway, and an alteration in protein kinase-C (PKC) activity [37]. Other diabetes-induced metabolic changes can also down-regulate the enzyme activity, including the increase in oxidative stress, the formation of advanced glycation products, the nerve growth factor metabolism [38], and the disturbance in essential fatty acid metabolism leading to an abnormal ω6/ω3 ratio in red blood cell membrane [39].

Moreover, insulin and C-peptide deficiencies can alter the long-term regulation of enzyme units expressed at the cell surface. The over-expression of α isoforms cannot be connected with an increase in enzyme activity; β isoforms are necessary to form an active complex. The C-peptide has short-term effect or NKA activity by modifying its phosphorylation status. Contrary to the down-regulation mechanisms, the mechanisms implicated in the NKA increase in some tissues of diabetic rat seems to be related to variations on mRNA levels of NKA isoforms. In rat skeletal muscle, insulin is able to produce a rapid translocation of preexisting a2 NKA from intracellular stores to the plasma membrane [40]. This results in the recruitment of additional functional Na pumps to the cell surface and increased NKA activity.

The impairment of NKA activity, mainly secondary to the lack of C-peptide, plays probably a role in the development of diabetic complications. The diabetes-induced decrease in enzyme activity would compromise microvascular blood flow by affecting microvascular regulation and decreasing red blood cell deformability, which lead to increased blood viscosity. C-peptide infusion restored red blood cell deformability and microvascular blood flow concomitantly with NKA activity [41]. In 2000, the same group had reported that insulin and C-peptide directly act on erythrocyte NKA, restoring the decreased tissue NKA activity observed in type-1 diabetic patients [42]. Low C-peptide level is considered to be responsible for low NKA activity in the red blood cells. Short-term C-peptide infusion to type 1 diabetic patients restores normal NKA activity. Islet transplantation, which restores endogenous C-peptide secretion, enhances NKA activity proportionally to the rise in C-peptide. This C-peptide effect is not indirect. In fact, incubation of diabetic red blood cells with C-peptide at physiological concentration leads to an increase of NKA activity. In isolated proximal tubules of rats or in the medullary thick ascending limb of the kidney, C-peptide stimulates in a dose-dependent manner NKA activity. The defect in ATPase is strongly related to diabetic neuropathy. Patients with neuropathy have lower ATPase activity than those without. The diabetes-induced impairment in NKA activity is identical in red blood cells and neural tissue. Red blood cell ATPase activity is related to nerve conduction velocity in the peroneal and the tibial nerve of diabetic patients. C-peptide infusion to diabetic rats increases endoneural ATPase activity in rat. Because the defect in NKA activity is also probably involved in the development of diabetic nephropathy and cardiomyopathy, physiological C-peptide infusion could be beneficial for the prevention of diabetic complications [41].

In order to elucidate the causal relationship between NKA and diabetic nephropathy, the erythrocyte enzyme activity was studied in type-2 diabetic patients (with microalbuminuria and without microalbuminuria) and in control subjects. NKA activity was significantly reduced in diabetic patients with hypertension and in microalbuminuric patients who had higher systolic BP and greater frequency of parental hypertension than those without microalbuminuria. Moreover, the enzyme activity in diabetic patients with parental hypertension was significantly reduced [43]. Contrary to this, in other research groups, the NKA activity was found to be higher in the diabetic than in the normal group, but the number of Na-pumps was not significantly different [44,45]. This increased activity of erythrocyte Na-pump in diabetes mellitus might suggest an increase of cation permeability associated with a possible disorder in the diabetic membrane. Conversely, the Na pumping activity (estimated from both NKA and ouabain-binding) was observed by others to be significantly decreased in type-1 and type-2 diabetic patients and to retain the insulin sensitivity only in young type-1 diabetics [46]. A significant reduction of NKA activity in erythrocyte membranes of type-1 diabetic patients, compared with matched controls, with similar contents of erythrocyte Na^+^ and K^+^ ion was also reported; when erythrocyte membranes of diabetic patients were incubated with their own plasma (probably containing higher concentration of a specific activator of NKA enzyme), a significant increase was found in enzyme activity, this effect being not influenced by the metabolic control [47]. Rabini and co-workers [48] observed that the structure of erythrocyte membranes, obtained from type-1 diabetic patients, showed a uncompetitive inhibition of the NKA, linked to the presence of conformational modifications of the protein. Modifications of the interactions between the enzymatic subunits and the membrane lipid environment might be at the basis of the NKA alteration in diabetes. With a microcalorimetric study, which allowed a direct measurement of the NKA activity in living red blood cells, a reduced Na, K-pump activity in diabetic patients were reported with a slower velocity of response to ouabain [49]. In type 1 diabetic patients, supplementation of C-peptide was shown to improve endothelium dependent vasodilatation in an NO-dependent pathway in different vascular compartments. In addition, it was observed that C-peptide administration in type 1 diabetic patients resulted in a redistribution of skin blood flow by increasing nutritive capillary blood flow in favor to subpapillary blood flow. Impaired NKA in another feature of diabetes mellitus in many cell types and is believed to be a pivotal regulator of various cell functions. C-peptide supplementation has been shown to restore NKA activity in different cell types during in vitro and in vivo investigations. In type 1 diabetic patients, C-peptide supplementation was shown to increase erythrocyte NKA activity by about 100%. A linear relationship was found between plasma C-peptide levels and erythrocyte NKA activity. In small capillaries, microvascular blood flow was increasingly determined by the rheologic properties of erythrocytes. Using laser-diffractoscopy a huge improvement in erythrocyte deformability was observed after C-peptide administration in erythrocytes of type 1 diabetic patients. Inhibition of the NKA by ouabain completely abolished the effect of C-peptide on erythrocyte deformability. Conclusively, C-peptide improves microvascular function and blood flow in type 1 diabetic patients by interfering with vascular and rheological components of microvascular blood flow [50].

### NKA correlation with hypertension, impact on salt balance and vascular contractility

The sodium pump is the only known receptor for the cardiac glycosides used to treat congestive heart failure and cardiac arrhythmias. This suggests that endogenous ligands structurally similar to cardiac glycosides may act as natural regulators of the sodium pump in heart and other tissues. Identification of naturally occurring regulators of NKA could initiate the discovery of new hormone-like control systems involved in the etiology of selected disease processes, hence the importance of understanding the relation of the sodium pump and its ligands to disease. The NKA contains a binding site for cardiac glycosides, such as ouabain, digoxin, and digitoxin, which is highly conserved among species ranging from Drosophila to humans.

Although advantage has been taken of this site to treat congestive heart failure with drugs such as digoxin, it is unknown whether this site has a natural function in vivo. However, this site plays an important role in the regulation of blood pressure, and it specifically mediates adrenocorticotropic hormone (ACTH)-induced hypertension in mice. Dostanic-Larson et al [51] used genetically engineered mice in which the NKA α2 isoform, which is normally sensitive to cardiac glycosides, was made resistant to these compounds. Chronic administration of ACTH caused hypertension in mice but not in mice with an ouabain-resistant α2 isoform of NKA. This finding demonstrates that the cardiac glycoside binding site of the NKA plays an important role in blood pressure regulation, most likely by responding to a naturally occurring ligand. Because the α1 isoform is sensitive to cardiac glycosides in humans, they developed mice in which the naturally occurring ouabain-resistant a1 isoform was made ouabain-sensitive. Mice with the ouabain-sensitive “human-like” α1 isoform and an ouabain-resistant α2 isoform developed ACTH-induced hypertension to greater extent than control animals. This result indicates that the cardiac glycoside binding site of the α1 isoform can also mediate ACTH-induced hypertension. Conclusively, these data provide conclusive evidence that the cardiac glycoside binding site, which mediates the pharmacological effects of digitalis and related drugs used in the treatment of congestive heart failure, is also the receptor for endogenous ligands involved in the regulation of cardiovascular function in vivo. Such results support the hypothesis that a steroid hormone may exist that regulates blood pressure through the interaction with the NKA.

The genetic and environmental heterogeneity of essential hypertension is responsible for the individual variability of antihypertensive therapy. An understanding of the molecular mechanisms underlying hypertension and related organ complications is a key aspect for developing new, effective, and safe antihypertensive agents able to cure the cause of the disease. Two mechanisms, among others, are involved in determining the abnormalities of tubular Na^+^ reabsorption observed in essential hypertension: the polymorphism of the cytoskeletal protein alpha-adducin and the increased circulating levels of endogenous ouabain (EO). Both lead to increased activity and expression of the renal Na^+^-K^+^ pump, the driving force for tubular Na transport. Morphological and functional vascular alterations have also been associated with EO. Rostafuroxin (PST 2238) is a new oral antihypertensive agent able to selectively antagonize EO, adducin pressor and molecular effects. It is endowed with high potency and efficacy in reducing blood pressure and preventing organ hypertrophy in animal models representative of both adducin and EO mechanisms. At molecular level, in the kidney, Rostafuroxin antagonizes EO triggering of the Src-epidermal growth factor receptor (EGFr)-dependent signaling pathway leading to renal Na^+^-K^+^ pump, and ERK tyrosine phosphorylation and activation. In the vasculature, it normalizes the increased myogenic tone caused by nanomolar ouabain. A very high safety ratio and an absence of interaction with other mechanisms involved in blood pressure regulation, together with initial evidence of high tolerability and efficacy in hypertensive patients, indicate Rostafuroxin as the first example of a new class of antihypertensive agents designed to antagonize adducin and EO-hypertensive mechanisms [52].

Weidmann and Ferrari have reported [53] that not only do type I (insulin-dependent) diabetic individuals have a familial predisposition for essential hypertension, but also normotensive offspring of parents who are non-diabetic but have essential hypertension show increased concentrations of plasma insulin and reduced insulin sensitivity; moreover, Na^+^ retention is characteristic of both type I and type II (non-insulin-dependent) diabetics. These authors also report that intracellular calcium is increased in adipocytes, in part via insulin's inhibition of Ca^2+^, Mg^2+^-ATPase, and that insulin may increase renal sodium retention and influence the activity of transmembrane electrolyte pumps. Insulin regulation of vascular NKA gene expression has been reported [54] as an important factor in the development of hypertension in diabetes. The mRNA encoding both the α1 and α2 isoforms was identified in vascular smooth muscle cells derived from embryonic rat thoracic aorta. Although the predominant isoform was α1, only the concentrations of the α2 isoform increased in response to insulin treatment. The overall increase in ouabain-inhibitable NKA activity in vascular smooth muscle cells in response to insulin treatment suggests that, in the absence of insulin or in insulin-resistant states, NKA activity could decrease, resulting in increased vascular contractility and blood pressure.

Renal NKA activity is bidirectionally regulated by natriuretic and antinatriuretic hormones, and a shift in the balance between these forces may lead to salt retention and hypertension. Dopamine plays a key role in this interactive regulation. By inhibiting vascular NKA activity, an excess of circulating ouabain may increase calcium concentration in vascular cells and lead to increased vascular contractility. Finally, mutations in cytoskeleton proteins may stimulate renal NKA activity by way of protein/protein interaction and lead to salt retention and hypertension [55]. Further, that the sodium transporters and NKA are segregated in membrane lipid and non-lipid raft microdomains that regulate their activities and trafficking via cytoskeletal proteins. The increase in renal proximal tubule ion transport in polygenic hypertension is primarily due to increased activity of NHE3 (sodium hydrogen exchanger isoform 3) and Cl/HCO3 exchanger at the luminal/apical membrane and a primary or secondary increase in NKA activity.   The increase in renal proximal tubule ion transport in hypertension is due to increased actions by prohypertensive factors that are unopposed by antihypertensive factors [56].

### Alterations in NKA activity in cardiovascular and renal disorders

The dysregulation of chief electrolytes especially sodium, potassium and calcium has a characteristic role in the cardiovascular and renal diseases. There occurs altered sodium and potassium concentrations in cardiovascular and renal patients primarily due to impaired NKA activity. Alterations take place in erythrocyte and plasma ionic environment in cardiovascular and renal patients. Under physiological conditions, NKA pump is the principal transporter, accounting for 1.4-2.0 mmol/rbc/hr. The Na^+^, K^+^-cotransport and sodium leak pathway are each responsible for approximately 0.2 mmol/rbc/hr. Most of the studies indicate that elevation of intracellular sodium and potassium was associated with a reduced activity of erythrocyte NKA pump [57]. Inhibition of NKA activity is the main factor as in most of the cardiovascular diseases, inhibited or reduced ATPase activity has been observed. The decreased serum sodium and increased serum potassium concentrations in renal patients have also been reported indicating that hyperkalemic state might have developed from a shift of potassium from intracellular to extracellular compartment or it could have been secondary to decreased renal potassium excretion. This increased potassium could also result from decreased renin production, which affects the aldosterone synthesis due to adrenal defect, which then would produce renal tubular secretory defect leading to abnormal distribution of potassium between intra and extracellular compartments [58].

In renal tubular epithelial cells, the expression and function of α1/β1 isoforms of the NKA are key in determining sodium ion reabsorption from the glomerular filtrate. Variable levels of endogenous sodium pump inhibitors seem to regulate this reabsorption, both by inhibiting enzymatic function and through altering plasmalemmal expression, and, thus, regulate renal sodium handling. The sodium pump also participates in the maintenance of intracellular sodium concentrations necessary for smooth muscle cell and cardiac myocyte functions [27]. Further, that the cardiotonic steroids (CTS) are essential participants in this regulation. In the classic or “ionic” signaling pathways for sodium pump function, it is quite clear how the inhibited sodium pump, coupled to the Na^+^/Ca^2+^-exchanger, may cause an increase in intracellular sodium, which in turn may lead to an increase in cytosolic calcium, the latter being a key second messenger for a variety of cell functions. The three classic features of NKA (the pump, the enzyme, and the receptor to cardiotonic steroids) are being understood in substantially greater detail, and, in some aspects, have undergone a paradigm shift in understanding. In particular, there is evidence that extremely low concentration of cardiotonic steroids, which are unlikely to inhibit the enzymatic function of the sodium pump [59,60], are able to initiate several signaling pathways, which may be extremely important for a variety of cell functions.

One single mechanism of the interaction of cardiac glycosides with the Na^+^ pump cannot explain the complexity of cellular responses resulting in cardiac inotropy, arterial hypertension, and remodeling of the circulatory system in association with tissue proliferation, as well as apoptotic processes. The well-known Na^+^-lag hypothesis, which assumes that inhibition of the a2-isozyme of NKA leads to an increase in Ca^2+^concentration, may explain short-term and inotropic actions of CTS. However, long-term effects leading to activation of genes and resulting in activation of cell proliferation, as well as apoptotic processes, are better described by the NKA signalosome hypothesis, which involves specific activation of signal transduction machinery. Depending on the gene expression pattern of the target cell, endogenous and exogenous cardiac glycosides may induce different physiological responses affecting not only the physiological action of cells of the circulatory system and an organism’s salt metabolism but, also, the growth of cancer cells. Cardiotonic steroids (CTS), long used to treat heart failure, are endogenously produced in mammals. Among them are the hydrophilic cardenolide ouabain and the more hydrophobic cardenolide digoxin, as well as the bufadienolides, marinobufagenin and telecinobufagin. Bufalin and oleandrin or the cardenolide analog UNBS-1450 block tumor cell proliferation and induce apoptosis at low concentrations in tumors with constitutive activation of NF-kappaB [61].

The NKA actively transports Na^+^ out and K^+^ into the myocyte. It is the receptor for cardiac glycosides exerting its positive inotropic effect by inhibiting enzyme activity, decreasing the driving force for the Na^+^/Ca^+^-exchange and increasing cellular content and release of Ca^+^ during depolarization. The specific binding capacity for cardiac glycosides is utilized as a tool for NKA quantification with high accuracy and precision. In treatment of patients with heart failure cardiac glycosides improve symptoms and reduce the need for hospitalization without affecting mortality. In endomyocardial biopsies from patients with compromised cardiac function total NKA concentration is decreased by about 40% and a correlation between decrease in heart function and decrease in NKA concentration exists. At the subunit level, the a1-, a3- and b1-proteins are reduced in human heart failure. During digitalization about 30% of remaining Na^+^, K^+^-pumps are occupied by digoxin. Thus, a total of not less than half the Na^+^, K^+^-pumps may be out of function in the myocardium of digitalized heart failure patients. It is still a matter of debate whether a digitalis-like factor exists. There is a pressing need for the identification of its precise chemical structure, properties and quantitative relation to the NKA. It is recommended that cardiac glycosides are prescribed to heart failure patients who are still having heart failure symptoms after institution of mortality reducing therapy. Cardiac glycoside treatment is still the only safe inotropic drug for oral use that improves hemodynamics in patients with compromised cardiac function [62].

Myocardial NKA is also influenced by other drugs used for the treatment of heart failure. Thus, potassium loss during diuretic therapy has been found to reduce myocardial NKA, whereas angiotensin-converting enzyme inhibitors may stimulate Na/K pump activity. Furthermore, hyperaldosteronism induced by heart failure has been found to decrease NKA activity. Accordingly, treatment with the aldosterone antagonist, spironolactone, may also influence NKA activity. The importance of Na/K pump modulation with heart disease, inhibition in digitalization and other effects of medication should be considered in the context of sodium, potassium and calcium regulation. It is recommended that digoxin be administered to heart failure patients who, after institution of mortality-reducing therapy, still have heart failure symptoms, and that the therapy is continued if symptoms are revealed or reduced. Digitalis glycosides are the only safe inotropic drugs for oral use that improve hemodynamics in heart failure. An important aspect of myocardial Na/K pump affection in heart disease is its influence on extra cellular potassium K_E_ homeostasis. Two important aspects should be considered: potassium handling among myocytes, and effects of potassium entering the extracellular space of the heart via the bloodstream. It should be noted that both of these aspects of K_E_ homeostasis are affected by regulatory aspects, for example, regulation of the Na/K pump by physiological and pathophysiological conditions, as well as by medical treatments. Digitalization has been shown to affect both parameters. Furthermore, in experimental animals, potassium loading and depletion are found to significantly affect K_E_ handling. The effects of potassium depletion are of special interest because this condition often occurs in patients treated with diuretics. In human congenital long QT syndrome caused by mutations in genes coding for potassium channels, exercise and potassium depletion are well known for their potential to elicit arrhythmias and sudden death. There is a need for further evaluation of the dynamic aspects of potassium handling in the heart, as well as in the periphery. It is recommended that resting plasma potassium be maintained at around 4 mM/L [63]. There is a tissue-specific regulation of NKA isoform expression in humans, as well as a highly specific regulation of the isoforms during disease, for example heart failure. There is also evidence for specific biochemical properties of different isoforms of the human NKA as well as for a specific functional impact on cardiac contractility in mice. Therefore, the isoforms of human NKA are not exchangeable and targeting specific isoforms by drugs or gene therapy may promise therapeutic benefit in diseases like heart failure or atrial fibrillation [64].

Myocardial infarction leads to compensatory ventricular remodeling. Disturbances in myocardial contractility depend on the active transport of Ca^2+^ and Na^+^, which are regulated by NKA. Inappropriate regulation of NKA activity leads to excessive loss of K^+^ and gain of Na^+^ by the cell. The NKA hypoactivity may modify the Na^+^, K^+^ and Ca^2+^ transport across the sarcolemma resulting in ventricular dysfunction [65]. In 2004, Dostanic et al have demonstrated that the a1 isoform of NKA regulates cardiac contractility, and that both the a1 and a2 isoforms are functionally and physically coupled with the Na/Ca exchanger in heart [66]. Intracellular sodium concentration ([Na^+^]_i_) modulates cardiac contractile and electrical activity through Na/Ca exchange (NCX). Upregulation of NCX in heart failure (HF) may magnify the functional impact of altered [Na^+^]_i_. Myocyte [Na^+^]_i_ is elevated in HF as a result of higher diastolic Na^+^ influx (with unaltered NKA characteristics). In HF, the combined increased [Na^+^]i, decreased Ca^2+^ transient, and prolonged action potential all profoundly affect cellular Ca^2+^ regulation, promoting greater Ca^2+^ influx through NCX during action potentials. Notably, the elevated [Na^+^]i may be critical in limiting the contractile dysfunction observed in HF [67]. The same research unit later reported that cardiac NKA regulates intracellular Na^+^, which in turn affects intracellular Ca^2+^ and contractility via the Na^+^/^2+^ exchanger. Extracellular K^+^ concentration ([K^+^]) is a central regulator of NKA activity. Phospholemman (PLM) has recently been recognized as a critical regulator of NKA in the heart. PLM reduces the intracellular Na^+^ affinity of NKA, an effect relieved by PLM phosphorylation. It was tested whether the NKA α1 vs. α2-isoforms have different external K^+^ sensitivity and whether PLM and PKA (Protein Kinase A) activation affects the NKA affinity for K^+^ in mouse cardiac myocytes. And measured the external [K^+^] dependence of the pump current generated by the ouabain-resistant NKA isoform in myocytes from wild-type (WT) mice (that is, current due to NKA -α1) and mice in which the NKA isoforms have swapped ouabain affinities (α1 is ouabain sensitive and α2 is ouabain resistant) to assess current due to NKA -α2. It was found that (1) NKA -α1 has higher affinity for K^+^ than NKA -α2 in cardiac myocytes; (2) PLM decreases the apparent external K^+^ affinity of NKA; and (3) phosphorylation of PLM at the cytosolic domain does not alter apparent extracellular K^+^ affinity of NKA [68].

Regarding isoform regulation, it is hypothesized that a primary decrease in cardiac NKA expression would be associated with a secondary increase in cardiac Na^+^/Ca^++^ exchanger (NCX) expression as a homeostatic mechanism to blunt an increase in cell Ca^++^ stores (and vice-versa with an increase in NKA). Supporting the hypothesis: in a rat model of renovascular hypertension, or after treatment with amiodarone there are 50% decreases in a-2 levels with 35-40% increases in NCX levels in left ventricle, while in the transition from hypo- to hyperthyroid, there are increases in both α-1 (2-fold) and α-2 (8-fold) with decreases in NCX (0.45-fold). In comparison, in transgenic mice over expressing NCX, there was no secondary change in NKA α-1 or α-2 levels indicating that primary changes in NCX do not drive secondary changes in NKA in the heart. This information provides the basis for addressing the significant gaps in our understanding of the physiologic, structural and homeostatic coupling between sodium pump isoforms and Na^+^/Ca^++^ exchangers in the heart and how coupling is related to control of cardiac contractility in health and disease [69].

### Regulatory role of nitric oxide on NKA in hypertension

One of the complications accompanying hypertension is the increase of intracellular concentration of sodium ions in the cardiac tissue and intracellular Na^+^ homeostasis is based on a balance between the influx and efflux of Na^+^. For the efflux of excessive Na^+^ ions out from the cells is responsible the NKA. It has been reported that hypertension was followed by remodeling of the Na^+^-binding site of the NKA molecule in the heart as an adaptation to enhanced [Na^+^]i induced by increased blood pressure, independently on the level of NO synthesis. On the other hand, the elevated activity of NO synthase induced an improvement of the ATP-affinity of the enzyme resulting thus in higher efficiency for utilizing the substrate ATP. In a situation of both effects, the hypertension and elevated activity of NO synthase occurred simultaneously, the influence of elevated activity of NO synthase seems to have a predominant role in regulating the function of cardiac NKA [70].

### Oxygen-induced regulation of NKA

The changes in oxygen availability render the adjustment of the NKA activity which is a matter of survival for neuronal cells. Petrushanko et al used freshly isolated rat cerebellar granule cells to study oxygen sensitivity of the NKA function. Along with transport and hydrolytic activity of the enzyme they have monitored alterations in free radical production, cellular reduced glutathione, and ATP levels. Both active K^+^ influx and ouabain-sensitive inorganic phosphate production were found to be maximal within the physiological pO_2_ range of 3-5 kPa. Transport and hydrolytic activity of the NKA was equally suppressed under hypoxic and hyperoxic conditions. The ATPase response to changes in oxygenation was isoform specific and limited to the a1-containing isozyme whereas a 2/3-containing isozymes were oxygen insensitive. Rapid activation of the enzyme within a narrow window of oxygen concentrations did not correlate with alterations in the cellular ATP content or substantial shifts in redox potential but was completely abolished when NO production by the cells was blocked by l-NAME (Nω-nitro-l-arginine) . Taken together their observations suggest that NO and its derivatives are involved in maintenance of high NKA activity under physiological conditions [71].

### NKA regulates sperm capacitation

In 2009, Newton et al observed that NKA was involved in the regulation of tyrosine phosphorylation in sperm proteins through receptor tyrosine kinase, non-receptor type protein kinase, and protein kinases A and C. It was inferred that the inhibition of NKA induced tyrosine phosphorylation and capacitation through multiple signal transduction pathways, imparting fertilizing ability in bovine sperm. It is an interesting report documenting both the involvement of ATP1A4 in the regulation of bovine sperm capacitation and that fresh bovine sperm capacitated by the inhibition of NKA can fertilize oocytes in vitro [72]. NKA acts as a signaling molecule during bovine sperm capacitation. Binding of ouabain to NKA inhibited motility (decreased progressive motility, average path velocity, and curvilinear velocity) and induced tyrosine phosphorylation and capacitation but did not increase intracellular calcium levels in spermatozoa. Furthermore, binding of ouabain to NKA induced depolarization of sperm plasma membrane. Therefore, binding of ouabain to NKA induced sperm capacitation through depolarization of sperm plasma membrane and signaling via the tyrosine phosphorylation pathway without an appreciable increase in intracellular calcium. Thundathil et al claimed in 2006, such finding to be the first report concerning the signaling role of NKA in mammalian sperm capacitation [73].

### Cell volume regulation through NKA

Excessive alterations of cell volume must be avoided for the survival of human and animal cells. The osmolarity amassed by cellular accumulation of organic substances must be compensated by lowering cytosolic ion concentrations. The Na^+^, K^+^ ATPase (NKA) extrudes Na^+^ in exchange for K^+^, which can permeate the cell membrane through K^+^ channels. K^+^ exit generates a cell-negative potential difference across the cell membrane, driving the exit of anions such as Cl^-^. The low cytosolic Cl^-^ concentrations counterbalance the excess cellular osmolarity by organic substances. Cell volume regulation following cell swelling involves releasing ions through activation of K^+^ channels and/or anion channels, KCl-cotransport, or parallel activation of K^+^/H^+^ exchange and Cl^-^/HCO^3^- exchange. Cell volume regulation following cell shrinkage involves accumulation of ions through activation of Na^+^, K^+^, 2Cl^-^ co-transport, Na^+^/H^+^ exchange in parallel to Cl-/HCO_3_^-^ exchange, or Na^+^ channels. The Na^+^ taken up is extruded by the NKA in exchange for K^+^. Shrunken cells further accumulate organic osmolytes such as sorbitol and glycerophosphorylcholine, and monomeric amino acids by altered metabolism and myoinositol (inositol), betaine, taurine, and amino acids by Na^+^ coupled transport. They release osmolytes during cell swelling. Challenges of cell volume homeostasis include transport, hormones, transmitters, and drugs. Moreover, alterations of cell volume participate in the machinery regulating cell proliferation and apoptotic cell death. Deranged cell volume regulation significantly contributes to the pathophysiology of several disorders such as liver insufficiency, diabetic ketoacidosis, hypercatabolism, fibrosing disease, sickle cell anemia, and infection [74].

### NKA involvement in ionic regulation of apoptosis

The loss of cell volume, chromatin condensation, internucleosomal DNA fragmentation, and apoptotic body formation are the distinct features of the apoptosis, an active process.  Among the classical characteristics that define apoptosis, the loss of cell volume has become a very important component of the programmed cell death process. Changes in cell volume result from alterations in the homeostasis of ions and in particular the movement of Na^+^ and K^+^ ions. Most living cells have a high concentration of intracellular K^+^ and a low concentration of intracellular Na^+^. This is in contrast to the outside of the cell, where there is a high concentration of extracellular Na^+^ and a low concentration of extracellular K^+^. Thus, a concentration gradient exists for the loss and gain of intracellular K^+^ and Na^+^, respectively. This gradient is maintained through the activity of various ionic channels and transporters, but predominantly the activity of the Na^+^, K^+^ ATPase (NKA). During apoptosis, there is compelling evidence indicating an early increase in intracellular Na^+^ followed by a decrease in both intracellular K^+^ and Na^+^ suggesting a regulatory role for these cations during both the initial signalling, and the execution phase of apoptosis. Recent studies have shown that the NKA is involved in controlling perturbations of Na^+^ and K^+^ homeostasis during apoptosis, and that anti-apoptotic Bcl-2 and Bcl-XL molecules influence these ionic fluxes. Finally, understanding the regulation or deregulation of ionic homeostasis during apoptosis is critical to facilitate the treatment of cardiovascular, neurological, and renal diseases where apoptosis is known to play a major role [75].

### NKA activity in rheumatoid arthritis patients

The NKA activity was significantly lower in Rheumatoid Arthritis (RA) patients than in both healthy controls and patients with osteoarthritis or gout. A slight but significant increase in Na_i_ was observed in rheumatoid subjects. It is hypothesized that the decrease in the NKA activity in RA may be the result of a defective expression of membrane proteins, which is probably related to the altered cell sensitivity observed [76]. Kiziltunç et al have also reported decrease in NKA in rheumatoid arthritis patients [77].

### Sepsis correlated NKA activity

Hsieh et al have reported that during sepsis, membrane alterations occur and result in an increased cellular Na^+^ content. (Vmax and v) was significantly stimulated, possibly as a consequence of a secondary response to the elevated Na^+^ of cells. Both cellular Na^+^ and Vmax were correlated well with the severity of sepsis, suggesting that these altered transport parameters may reflect the progress of sepsis [78].

### NKA in relation to neurological disorders

The cytosol of nerve endings are reported to contain the factors regulating the activity of synaptosomal NKA. These factor stimulated by neurotransmitters activates the NKA system affecting the phosphorylating intermediates of the enzyme and putting the NKA system in the mode of simultaneous transport of Na and K ions [79]. Psychiatric abnormalities have been described in primary neurological disorders like multiple sclerosis, primary generalized epilepsy, Parkinson's disease, subacute sclerosing panencephalitis (SSPE), central nervous system glioma, and syndrome X with vascular dementia. In 2003, Kurup and Kurup considered pertinent to compare monoamine neurotransmitter pattern in schizophrenia with those in the disorders mentioned above. The end result of neurotransmission is changes in membrane NKA activity. Membrane NKA inhibition can lead to magnesium depletion, which can lead to an upregulated isoprenoid pathway. The isoprenoid pathway produces three important metabolites: digoxin; an endogenous membrane NKA inhibitor; ubiquinone, a membrane antioxidant and component of mitochondrial electron transport chain, and dolichol, important in N-glycosylation of protein. The serum/plasma levels of digoxin, dolichol, ubiquinone, magnesium, HMG CoA reductase activity, and RBC NKA activity were estimated in all these disorders. The result showed that the concentration of serum tryptophan and serotonin was high and serum tyrosine, dopamine, adrenaline, and nor-adrenaline low in all the disorders studied. The plasma HMG CoA reductase activity, serum digoxin, and serum dolichol levels were high and serum ubiquinone levels, serum magnesium, and RBC NKA activity were low in all these disorders [80].

### NKA associated with migraine pathophysiology

Cerebrospinal fluid sodium concentration ([Na^+^]_csf_) increases during migraine, but the cause of the increase is not known. In 2009, Harrington et al analyzed biochemical pathways that influence [Na^+^]_csf_ to identify mechanisms that are consistent with migraine and inferred that increased capillary endothelial cell (CEC) NKA transporter (NKAT) activity is probably the primary cause of increased [Na^+^]_csf_. Physiological fluctuations of all NKAT regulators in blood, many known to be involved in migraine, were monitored by receptors on the luminal wall of brain CECs; signals then transduced to their abluminal NKATs that alter brain extracellular sodium ([Na^+^]e) and potassium ([K^+^]_e_). They proposed a theoretical mechanism for aura and migraine when NKAT activity shifts outside normal limits: (1) CEC NKAT activity below a lower limit increases [K^+^]_e_, facilitates cortical spreading depression, and causes aura; (2) CEC NKAT activity above an upper limit elevates [Na^+^]_e_, increases neuronal excitability, and causes migraine; (3) Migraine-without-aura may arise from CEC NKAT over-activity without requiring a prior decrease in activity and its consequent spreading depression; (4) migraine triggers disturb, and treatments improve, CEC NKAT homeostasis; (5) CEC NKAT-induced regulation of neural and vasomotor excitability coordinates vascular and neuronal activities, and includes occasional pathology from CEC NKAT-induced apoptosis or cerebral infarction [81]. As mentioned above the NKA is an ion-translocating transmembrane protein that actively maintains the electrochemical gradients for Na^+^ and K^+^ across the plasma membrane. The functional protein is a heterodimer comprising a catalytic α-subunit (four isoforms) and an ancillary β-subunit (three isoforms). Mutations in the α2-subunit have recently been implicated in familial hemiplegic migraine type 2, but almost no thorough studies of the functional consequences of these mutations have been provided. However, in 2005 Koenderink et al [82] investigated the functional properties of the mutations L764P and W887R in the human NKA a2-subunit upon heterologous expression in Xenopus oocytes. No NKA-specific pump currents could be detected in cells expressing these mutants. The binding of radiolabelled [3H] ouabain to intact cells suggested that this could be due to a lack of plasma membrane expression. However, plasma membrane isolation showed that the mutated pumps are well expressed at the plasma membrane. 86Rb^+^-flux and NKA activity measurements demonstrated that the mutants are inactive. Therefore, the primary disease-causing mechanism is loss-of-function of the NKA α2-isoform.

### NKA expression, regulation and function in the lens

The required concentrations of sodium and potassium in lens cells are maintained by NKA. NKA activity is different in the two cell types that make up the lens, epithelial cells and fibers; specific activity in the epithelium is higher than in fibers. In some parts of the fiber mass NKA activity is barely detectable. Several reports suggest that NKA-mediated ion transport by the epithelium contributes significantly to the regulation of ionic composition in the entire lens. In some species different NKA isoforms are present in epithelium and fibers but in general, fibers and epithelium express a similar amount of NKA protein. The turnover of NKA by protein synthesis may contribute to preservation of high NKA activity in the epithelium. In ageing lens fibers, oxidation, and glycation may decrease NKA activity. NKA activity in lens fibers and epithelium also may be subject to regulation as the result of protein tyrosine phosphorylation. Moreover, activation of G protein-coupled receptors by agonists such as endothelin-1 elicits changes of NKA activity. The asymmetrical distribution of NKA activity in the epithelium and fibers may contribute to ionic currents that flow in and around the lens. Studies on human cataract and experimental cataract in animals reveal changes of NKA activity but no clear pattern is evident. However, there is a convincing link between abnormal elevation of lens sodium and the opacification of the lens cortex that occurs in age-related human cataract [83].

### Role of NKA in lung edema clearance

In 2002 Sznajder et al [84] have reported that acute hypoxemic respiratory failure is a consequence of edema accumulation due to elevation of pulmonary capillary pressures and/or increases in permeability of the alveolocapillary barrier. It has been recognized that lung edema clearance is distinct from edema accumulation and is largely affected by active Na^+^ transport out of the alveoli rather than reversal of the Starling forces, which control liquid flux from the pulmonary circulation into the alveolus. The alveolar epithelial NKA has an important role in regulating cell integrity and homeostasis. In the last 15 years, NKA has been localized to the alveolar epithelium and its contribution to lung edema clearance has been appreciated. The importance of the alveolar epithelial NKA function is reflected in the changes in the lung's ability to clear edema when the NKA is inhibited or increased. An important focus of the ongoing research is the study of the mechanisms of NKA regulation in the alveolar epithelium during lung injury and how to accelerate lung edema clearance by modulating NKA activity. Report from Matthay et al [85] emphasized that the rate of fluid transport can be increased in most species, including the human lung, by cAMP stimulation. Catecholamine-independent mechanisms, including hormones, growth factors, and cytokines, can also upregulate epithelial fluid clearance in the lung. The new insight into the role of the distal lung epithelium in actively regulating lung fluid balance has important implications for the resolution of clinical pulmonary edema.

### Hormonal regulation of NKA

The patients often complain of muscular weakness, exercise intolerance, cramps and excessive fatigability during thyroid deficiency. Hypothyroidism induces a metabolic myopathy, with a fall of the energetic production, and especially of the mitochondrial metabolism. This is due to a global inhibition of the main oxidative pathways (substrate incorporation, substrate oxidation) and of the respiratory chain. A diminished energetic consumption is partially related to a transition in the myosin isoforms, which express a slower ATPase, and to an impairment of the trans-sarcolemic transports. The decreased number of NKA dependent pumps could imply an abnormal intracellular Na^+^ level and explain the frequent disorders of the membrane excitability [86]. T3 (3, 3’, 5-triiodo-L-thyronine) plays an important role in cell survival and differentiation. Nongenomic regulation of phosphatidylinositol 3-kinase and downstream molecules by T3 is being recognized in different tissues. Upregulation of alveolar NKA is one such molecule, which plays an important role in removal of edema fluid from the alveolar space. These effects are rapid and do not require direct nuclear gene transcription [87]. In 2007, Phakdeekitcharoen et al have reported the first evidence that, in human skeletal muscles, thyroid hormone upregulates the NKA protein expression at least, in part, at mRNA level, and the a2 and b1 subunits play the important role in this regulation [88].

Aldosterone, a major regulator of Na transport by the collecting duct, stimulates NKA activity through both recruitment of intracellular pumps and increased total amounts of Na pump subunits. And we know that the collecting duct is the site of final sodium reabsorption according to Na balance requirements. Vasopressin and cAMP, its second messenger, stimulate NKA activity within minutes through translocation of sodium pumps from a brefeldin A-sensitive intracellular pool to the plasma membrane. Dysregulation of collecting duct NKA activity is at least in part responsible of the sodium retention observed in nephritic syndrome. Thus, aldosterone, vasopressin, and intracellular sodium control the cell surface expression of NKA and translocation from intracellular stores is a major mechanism of regulation of NKA activity in collecting duct principal cells [89].

In the kidney, the collecting duct (CD) is the site of final sodium reabsorption, according to Na^+^ balance requirements. In this segment of the renal tubule, principal cells may reabsorb up to 5% of the filtered sodium. The driving force for this process is provided by the basolateral NKA (sodium pump). NKA activity and expression in the CD are modulated physiologically by hormones (aldosterone, vasopressin, and insulin) and non-hormonal factors including intracellular (Na^+^) and extracellular osmolality [90].

In 2008, Kamenicky et al [91] observed that acromegalic GC rats (genetic catalepsy rats), which are chronically exposed to very high levels of GH (Growth Hormone), exhibited a decrease of furosemide-induced natriuresis and an increase of amiloride-stimulated natriuresis compared with Wistar rats (controls). Enhanced NKA activity and altered proteolytic maturation of epithelial sodium channel (ENaC) subunits in the cortical collecting ducts (CCDs) of GC rats provided additional evidence for an increased sodium reabsorption in the late distal nephron under chronic GH excess. They reported that GH directly controls sodium reabsorption in CCD cells is supported by: (1) stimulation of transepithelial sodium transport inhibited by GH receptor antagonist pegvisomant; (2) induction of alpha-ENaC mRNA expression; and (3) identification of signal transducer and activator of transcription 5 binding to a response element located in the alpha-ENaC promoter, indicative of the transcriptional regulation of alpha-ENaC by GH. Their findings provide the first evidence that GH, in concert with IGF-I, stimulates ENaC-mediated sodium transport in the late distal nephron, accounting for the pathogenesis of sodium retention in acromegaly.

### Role of NKA in preeclampsia

Preeclampsia is a disorder of pregnancy characterized by pregnancy-induced hypertension (≥140 mm Hg systolic and / or ≥ 90 mm Hg diastolic blood pressure), new-onset proteinuria (≥ 300 mg protein/day), and edema occurring in the second half of pregnancy. It is a disease characterized by hypertension and proteinuria but can manifest many abnormalities. We have earlier reported that preeclampsia involves the significant increase in the plasma concentrations of nitric oxide [92]. As mentioned above the elevated activity of NO synthase induced an improvement of the ATP-affinity of NKA resulting thus in higher efficiency for utilizing the substrate ATP. Some of the best documented alterations involve changes in the handling of sodium ion both on the systemic and on the cellular level. A majority of studies support an increase in peripheral cell sodium concentration. This would suggest a defect in NKA or sodium pump activity. Direct study of cellular sodium pump activity provides suggestive but not unequivocal support for this decreased sodium pump activity. Other evidence indicates increased circulating concentrations of a sodium pump inhibitor in most, but not all, studies of preeclampsia.  Together, current research argues more strongly in favor of derangements of cell sodium handling perhaps mediated by circulating sodium pump inhibitors leading often to increased cell sodium. Such an increase of cell sodium in vascular tissue has previously been shown to enhance vascular sensitivity to vasoconstrictor agents or lead directly to increased vasoconstriction [93].

### Role of NKA in carcinoma

In 2003, Rajasekaran et al have reported that NKA function is necessary for the formation and maintenance of tight junctions in epithelial cells, suggesting that NKA plays an essential role in regulating the transport and polarized phenotype of epithelial cells. Loss of the polarized phenotype of epithelial cells is a characteristic of carcinoma and correlates with their invasiveness and metastatic potential. Consistent with an important role for NKA in the regulation of polarized phenotype of epithelial cells, both NKA enzyme activity and subunit levels are reduced in carcinoma [94]. NKA-β levels were highly reduced in an invasive form of human renal clear cell carcinoma [94], androgen-dependent prostate cancer [95], in early stages of urothelial cancer, as well as in poorly differentiated, highly motile carcinoma cell lines obtained from various tissues [96], suggesting a functional link between reduced NKA-β expression and cancer progression. In contrast, the levels of NKA-α did not reveal a consistent pattern in different carcinomas. Increased cell motility is a prerequisite for invasion and metastasis, and in general most of the invasive carcinoma cell lines are highly motile.

Recently, it has been shown that NKA β localizes to lamellipodia and suppresses cell motility by a novel signaling mechanism involving a cross-talk between NKA α1-subunit (NKA α) and NKA β with proteins involved in phosphatidylinositol 3-kinase (PI3-kinase) signaling pathway. Further, that NKA α associates with the regulatory subunit of PI3-kinase and NKA β binds to annexin II. These molecular interactions locally activate PI3-kinase at the lamellipodia and suppress cell motility in MSV-MDCK cells, independent of NKA ion transport activity. Thus, these results demonstrate a new role for NKA in regulating carcinoma cell motility [97].

Earlier studies demonstrated altered NKA subunit expression in renal clear cell carcinoma and an association of subunit levels with the prediction of recurrent bladder cancer. In 2008, Seligson et al [98] determined the clinical association of protein expression patterns of the NKA α1 and β1-subunits in renal clear cell carcinoma using tissue microarrays with linked clinicopathological data. However, their results suggest that NKA α1-subunit expression patterns may be a useful clinical prognosticator for renal clear cell carcinoma. The NKA β1-subunit was not found to be a useful prognosticator in this setting.

The sodium pump (NKA) could be a target for the development of anticancer drugs as it serves as a signal transducer, it is a player in cell adhesion and its aberrant expression and activity are implicated in the development and progression of different cancers. Cardiotonic steroids (CTS), are the natural ligands and inhibitors of the sodium pump and this supports the possibility of their development as anticancer agents targeting overexpressed NKA α-subunits. In 2008, Mijatovic et al have emphasized that targeting over-expressed NKA α-subunits using novel CTS might open a new era in anticancer therapy and bring the concept of personalized medicine from aspiration to reality. Clinical data are now needed to further support this proposal. Furthermore, future medicinal chemistry should optimize new anticancer CTS to target NKA α-subunits with the aim of rendering them more potent and less toxic [99].
